# A Comprehensive Evaluation of NIPAM Polymer Gel Dosimeters on Three Orthogonal Planes and Temporal Stability Analysis

**DOI:** 10.1371/journal.pone.0155797

**Published:** 2016-05-18

**Authors:** Kai-Yuan Cheng, Ling-Ling Hsieh, Cheng-Ting Shih

**Affiliations:** 1 Department of Medical Imaging and Radiological Sciences, Central Taiwan University of Science and Technology, Taichung, Taiwan; 2 Graduate Institute of Pharmaceutical Science and Technology, Central Taiwan University of Science and Technology, Taichung, Taiwan; 3 3D Printing Medical Research Center, China Medical University Hospital, China Medical University, Taichung, Taiwan; Technische Universitaet Muenchen, GERMANY

## Abstract

Polymer gel dosimeters have been proven useful for dose evaluation in radiotherapy treatments. Previous studies have demonstrated that using a polymer gel dosimeter requires a 24 h reaction time to stabilize and further evaluate the measured dose distribution in two-dimensional dosimetry. In this study, the short-term stability within 24 h and feasibility of *N*-isopropylacrylamide (NIPAM) polymer gel dosimeters for use in three-dimensional dosimetry were evaluated using magnetic resonance imaging (MRI). NIPAM gels were used to measure the dose volume in a clinical case of intensity-modulated radiation therapy (IMRT). For dose readouts, MR images of irradiated NIPAM gel phantoms were acquired at 2, 5, 12, and 24 h after dose delivery. The mean standard errors of dose conversion from using dose calibration curves (DRC) were calculated. The measured dose volumes at the four time points were compared with those calculated using a treatment planning system (TPS). The mean standard errors of the dose conversion from using the DRCs were lower than 1 Gy. Mean pass rates of 2, 5, 12, and 24 h axial dose maps calculated using gamma evaluation with 3% dose difference and 3 mm distance-to-agreement criteria were 83.5% ± 0.9%, 85.9% ± 0.6%, 98.7% ± 0.3%, and 98.5% ± 0.9%, respectively. Compared with the dose volume histogram of the TPS, the absolute mean relative volume differences of the 2, 5, 12, and 24 h measured dose volumes were lower than 1% for the irradiated region with an absorbed dose higher than 2.8 Gy. It was concluded that a 12 h reaction time was sufficient to acquire accurate dose volume using the NIPAM gels with MR readouts.

## Introduction

Intensity-modulated radiation therapy (IMRT) has been widely applied in modern radiation therapy. Pretreatment verifications have become a crucial part of routine patient-specific quality control in IMRT [1]. Traditional measurement tools, such as ion chambers and films, have been used to verify the dose distribution of IMRT. However, these tools provide only point or planar dose measurements. To fully verify a three-dimensional (3D) dose distribution, Gore et al. [2] used a ferrous sulfate gel dosimeter (Fricke gel) to measure the dose distribution in three dimensions. However, the measured dose distribution is strongly influenced by readily dispersed ferric ions, resulting in low signals, blurred images, and, ultimately, errors in dose measurement. In 1958, Hoecker and Watkins reported that the critical diffusion of ferric ions in Fricke gel can be prevented using a radiation-induced polymerized monomer [3]. In the last decade, polymer gel dosimeters have become useful in measuring dose distribution. In contrast to traditional measurement tools, polymer gel dosimeters can capture the entire 3D dose distribution in a single measurement without signification diffusion. In addition, polymer gel dosimeters demonstrate the advantages of easy shaping and are equivalent to human tissues.

The basic physical process of polymer gel dosimetry relies on water radiolysis, leading to radicals that interact with monomers, thereby initializing the polymerization reaction. When the polymerization reaction is completed, the chains become spatially trapped in the parts of the gel matrix affected by radiation. Therefore, the dose distribution can be obtained by measuring the changes of growing polymer chains. Several modalities have been used, including X-ray computed tomography [4], optical computed tomography [5,6], ultrasound [7], and magnetic resonance imaging (MRI) [8,9]. In MRI, the spin–spin relaxation rate (R2) depends on the mobility of water molecules. The polymer chains formed in the gel matrix reduce the mobility of water molecules. MRI can therefore determine the degree of polymerization through T2-weighted imaging. Additionally, MRI demonstrates the advantages of high spatial resolution and no additional dose to gel.

Senden et al. [10] proposed a new polymer gel, mainly composed of gelatin, *N*-isopropylacrylamide (NIPAM), Bis, and tetrakis (hydroxymethyl) phosphonium chloride (THPC), with a high radiation sensitivity that allows the reaction of monomers and free radicals in the irradiated region. An increasing number of reports [11–13] show that NIPAM gel dosimeters have potential for use in the verification of radiotherapy dose distributions. Previous work [11,12] has focused on the fundamental characteristics of NIPAM polymer gel dosimeters and the feasibility of an NIPAM/MRI system for clinical 3D dosimetry. Gel dosimeters are generally considered stabilized and readable at 24 h postirradiation [11,13]. In subsequent investigations, simple dose distributions and γ-index maps from gel measurements have been compared with those from treatment planning system (TPS) calculations regarding central axial planar dose distributions [14].

In this study, the short-term stability and feasibility of the NIPAM gel dosimeter with MR readouts were evaluated using a clinical case of eye tumor intensity-modulated radiation therapy (IMRT). Dose maps and dose volume measured from the NIPAM gel dosimeters at 2, 5, 12, and 24 h postirradiation were compared to that calculated from a treatment planning system. Dose maps in axial, coronal, and sagittal views were evaluated using the isodose maps, dose profiles and gamma evaluation. In addition, the entire dose volume was evaluated uising dose volume histogram (DVH).

## Materials and Methods

### NIPAM gel dosimeter preparation

The gel used in this study consisted of 5% gelatin (300 Bloom Tape A, Sigma-Aldrich), 3% NIPAM (97%, Sigma-Aldrich), 3% BIS (Merck), 10 mM THPC (80%, Sigma-Aldrich), and 87% deionized water. The NIPAM polymer gel was prepared according to the instructions of Senden et al. [10]. After manufacture, the gels were poured into Pyrex tubes for calibration and into three cylindrical gel phantoms for dose distribution measurement; the tubes were subsequently placed in cylindrical polymethylmethacrylate phantom containers (130 mm diameter, 130 mm height, and 5 mm wall thickness) covered with aluminum foil. The containers were then stored in a refrigerator at 4°C to prevent light-induced prepolymerization until complete solidification was achieved. After dose delivery and between MR scans, the gel phantoms and calibration tubes were placed in the scanning room at 23 ± 1°C to reduce the influence of temperature on the polymerization reaction.

### Dose delivery

#### Calibration tubes

The NIPAM gels were irradiated using a 6 MV linear accelerator (Clinac 21EX LINAC, Varian Medical Systems, USA). Quality assurance of the linear accelerator was regularly performed, thereby passing the regulations of Taiwan. The photon output error of the medical accelerator was validated daily at lower than 3%. The irradiation was performed at the following settings: beam angle, 0°; dose rate, 4 Gy/min; and field size, 10 × 10 cm^2^. The dosimeters were placed in an acrylic phantom (length, 30 cm; width, 30 cm; thickness, 4 cm), placed between two 3 cm-thick solid water slabs. Seven calibration tubes were prepared to determine a dose response curve (DRC) for the dose conversion. The doses delivered to the tubes were 0, 1, 2, 5, 8, 10, and 12 Gy.

#### Cylindrical gel phantoms

For treatment planning, CT images of a cylindrical phantom were acquired to obtain the geometry by using a simulation CT (CT Simulation, Marconi AcQSim, Philips, UK). The cylindrical phantom was filled with gelatin to prevent unnecessary dose absorption in the gels and to mimic the gels’ photon attenuation characteristics. The CT images were imported into the TPS, and an IMRT plan for eye tumor treatment was generated using the Eclipse TPS v10.0 (Varian Medical Systems, Palo Alto, CA). Three cylindrical gel phantoms filled with the NIPAM gel were irradiated using dose delivery techniques identical to those commonly used in patient treatments. The irradiation condition settings were as follows: prescribed dose at isocenter, 5 Gy; photon beam energy, 6 MV; dose rate, 400 cGy/min; number of fields, five; and source-to-axis distance (SAD), 100 cm.

### MRI scanning and data analysis

**Fig 1:**
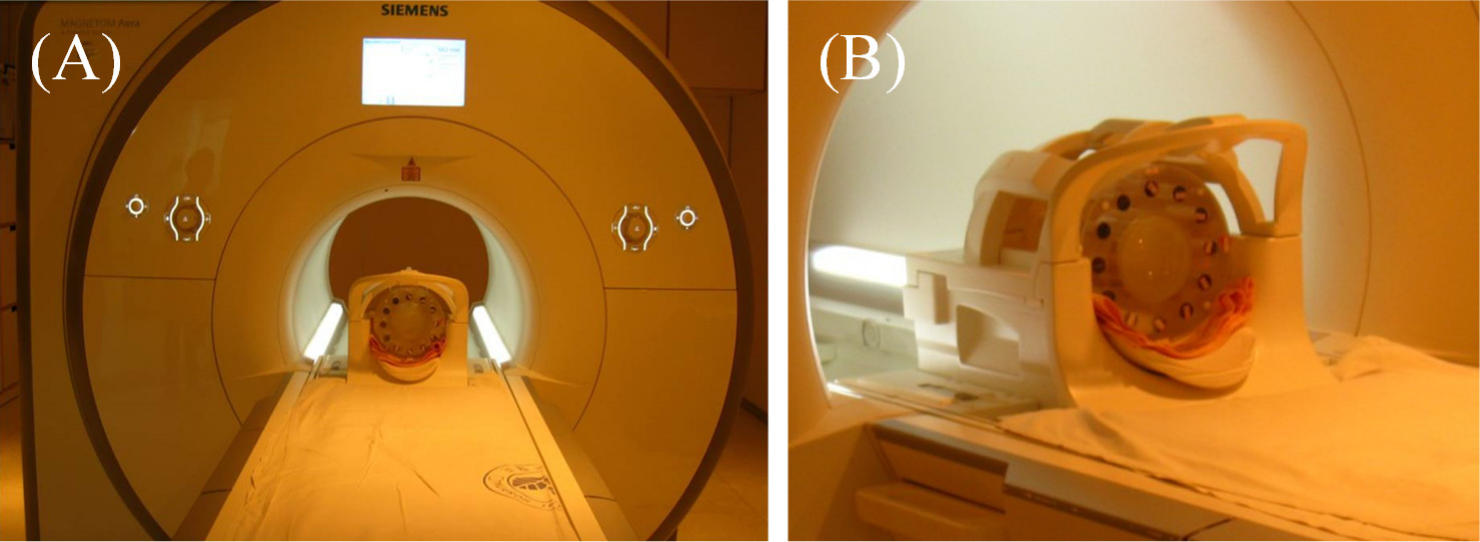
**(A) MRI scanner for dose readout of NIPAM polymer gel dosimeters.** (B) Scanning position of the customized acrylic holder in a head coil.

A clinical 1.5 Tesla MRI scanner (MAGNETOM Aera MRI Scanner, Siemens, Germany) with a head coil was used to scan the gel phantoms. As shown in Fig 1, the calibration tubes and gel phantoms were inserted into a customized acrylic holder. The T2-weighted images of the tubes and phantoms were acquired using a multiple-spin echo sequence with the following parameters: TR, 3000 ms; echo spacing, 22 ms; number of echoes, 16; FOV, 240×240 mm^2^; matrix size, 512×512; and slice thickness, 5 mm.

After scanning, the acquired MR images were analyzed using MATLAB (The MathWorks, Natick, MA, USA). The DRC was determined using the following three procedures. First, regions of interest were drawn in the tubes to measure the mean signal. Employing the many-points method [15], least-square fittings were performed to determine the T2 values of each tube, using the T2 relaxation model, mean signals, and echo times of 16 echoes. Second, R2 values were calculated by 1/T2 values. Finally, the DRC was determined by linear fitting the R2 values and the absorbed doses of the tubes by using R2 = *a* × Dose + *b*. Similarly, the MR images of the cylindrical gel phantom were converted to R2 maps by using the aforementioned procedures on a pixel-by-pixel basis. Using the DRC, the R2 maps were converted into dose maps directly. In addition, to investigate the time polymerization reaction characteristics of NIPAM gel under MRI, the calibration tubes and three gel phantoms were scanned at four time points: 2, 5, 12, and 24 h after dose delivery. To accurately obtain T2-weighted images at the four time points, the calibration tubes and three gel phantoms were separately irradiated with a 40 min interval for MR imaging. On the basis of the irradiation time, the T2-weighted images of the calibration tubes and three gel phantoms were separately acquired at the four time points. The R2 maps were converted into dose maps by using the DRCs obtained from the same time points.

### Evaluation

#### Uncertainty of dose conversion

To evaluate the errors from dose conversion, the uncertainty of the dose converted from the measured R2 maps by using the DRCs was calculated. Dose–response relationship, or calibration curve, is a widely recognized quantitative tool in science and technology. Typically, a single response measurement *y* is related to a single predictor *x* for each observation using a simple linear regression as follows:

$$y_{i}=\alpha \cdot x_{i}+\beta +\varepsilon_{i},$$ (1)

where *y*_*i*_ is the *i*th observation of the response to dose *x*_*i*_; *β* is the intercept; *α* is the slope; and *ε*_*i*_ is an unobservable error term with zero mean and constant variance *σ*^2^, i.e., {*ε*_*i*_: 1≦*i*≦*n*} is independent and identically normally distributed as *N*(0, *σ*^2^). In our study, response *y* is the relaxation rate *R*2 and the predictor *x* is dose *D*. We seek to fit the *n* data points (*D*_*i*_, *R*2_*i*_) into the linear model given as follows:

$$\overset{⌢}{R}2=a\cdot D+b,$$ (2)

where $\overset{⌢}{R}2$ be the prediction of *R*2, *a* is the least squares estimator of the slop, and *b* is the least squares estimator of the intercept. The standard error of $\overset{⌢}{R}2$ is the appropriate quantity to use for error bars on *R*2 values obtained from the best fit in the least squares. The standard error of $\overset{⌢}{R}2$ at *D* = *D*_*p*_ can be computed using the following formula [16]:

$$SE(\overset{⌢}{R}2)=s_{R2,D}\cdot \sqrt{\frac{1}{n}+\frac{{\left(D_{p}-\bar{D}\right)}^{2}}{\sum_{i=1}^{n}{\left(D_{i}-\bar{D}\right)}^{2}}},$$ (3)

where *s*_*R*2,*D*_ is the standard deviation of *R*2(*D*) and is defined by

$$s_{R2,D}=\sqrt{\frac{1}{n-2}\sum_{i=1}^{n}{\left(R2_{i}-\overset{⌢}{R}2_{i}\right)}^{2}}.$$ (4)

The inverse of this problem is a point of interest. In reverse regression, we regard *D* as the response and R2 as the predictor. In our study, the values (*R*2_*i*_, *D*_*i*_) comprise a set of *n* data pairs in which we wish to fit the reverse regression model. We express our estimated model [17] with

$$\overset{⌢}{D}=\beta +\alpha \left(R2-\bar{R}2\right),$$ (5)

where $\overset{⌢}{D}$ is the prediction of *D*, $\beta =\bar{D}$, and

$$\alpha =\frac{\sum_{i=1}^{n}\left(R2_{i}-\bar{R}2\right)\cdot \left(D_{i}-\bar{D}\right)}{\sum_{i=1}^{n}{\left(R2_{i}-\bar{R}2\right)}^{2}}.$$ (6)

This method disregards the simple linear regression assumption that the predictor is measured with a negligible error (Parker et al., 2010). For a future observed value of *R*2_0_, the standard error of *D*_0_ using reverse regression is as follows:

$$SE({\overset{⌢}{D}}_{0})=s_{D,R2}\cdot \sqrt{1+\frac{1}{n}+\frac{{\left(R2_{0}-\bar{R}2\right)}^{2}}{\sum_{i=1}^{n}{\left(R2_{i}-\bar{R}2\right)}^{2}}}\;,$$ (7)

where *s*_*D*,*R*2_ is the standard deviation of *D*(*R*2) and is defined by

$$s_{D,R2}=\sqrt{\frac{1}{n-2}\sum_{i=1}^{n}{\left(D_{i}-{\overset{⌢}{D}}_{i}\right)}^{2}}.$$ (8)

In our study, the standard errors of the parameters *a* and *b* (Δ*a* and Δ*b*), and the mean standard errors of the dose conversion from using the DRCs (mean Δ*D*) were calculated for each measured time point.

#### Evaluation of measured dose distribution

In this study, the gamma evaluation proposed by Low et al. [18] was used to evaluate the dose maps measured using NIPAM gel with MR readouts. The gamma evaluation comprises both dose difference and distance-to-agreement (DTA) comparison criteria for comparing two dose maps. The gamma index quantitatively represents the difference between dose maps and can be calculated using Eqs 1,2,3 and 4.

$$\gamma \left(r_{m}\right)=\text{min}\left\{\Gamma \left(r_{m},r_{c}\right)\right\}\forall \left\{r_{c}\right\},$$ (9)

where Γ is a gamma function and is described as follows:

$$\Gamma \left(r_{m},r_{c}\right)=\sqrt{\frac{d^{2}\left(r_{m},r_{c}\right)}{\Delta d_{M}^{2}}+\frac{\delta^{2}\left(r_{m},r_{c}\right)}{\Delta D_{M}^{2}}},$$ (10)

where

$$r\left(r_{m},r_{c}\right)=\left|r_{c},r_{m}\right|$$ (11)

and

$$\delta \left(r_{m},r_{c}\right)=D_{m}\left(r_{m}\right)-D_{c}\left(r_{c}\right),$$ (12)

where *r*_*m*_ and *r*_*c*_ are the spatial locations of the dose pixels in the measured and calculated dose maps, respectively. The terms *d* and *δ* respectively denote the spatial distance and dose difference between pixels, and Δ*d*_*M*_ and Δ*D*_*M*_ represent the DTA and dose difference comparison criteria, correspondingly. A pixel with a gamma value lower than 1 indicates that the combined errors of the spatial distance and dose difference of the pixel were lower than the predefined criteria, thus passing the evaluation. By contrast, a pixel with a gamma index greater than 1 corresponds to the locations where the measured value does not satisfy the criteria. Finally, the pass rate of a gamma map was calculated using the number of passed pixels divided by the pixel number of the gamma map and multiplied by 100%. The pass rate therefore represents the percentage of a dose map satisfying the evaluation criteria. In this study, the dose maps measured using NIPAM gels were compared with those from the TPS by using the criteria of a 3% dose difference (Δ*D*_*M*_) and 3 mm DTA (Δ*d*_*M*_), which are the most frequently used criteria in published comparisons of treatment plans [19].

In addition to the gamma evaluation, DVH, which is generally used to analyze the 3D dosimetry and quality of treatment plans in clinical practice, was calculated to compare the dose volumes of NIPAM gel and TPS. The relative volume (RV) covered by an absorbed dose was calculated from 0% to 100% of the prescribed dose with an interval of 1%. The percent RV differences between the dose volumes from the TPS and the measurements across a dose range from an absorbed dose starting at 0 Gy to the prescribed dose were also calculated using the following equation:

$$d_{RV}\%=\frac{RV_{TPS}\%\left(D_{s},\;D_{p}\right)-RV_{m,t}\%\left(D_{s},\;D_{p}\right)}{RV_{m}\%\left(D_{s},\;D_{p}\right)},$$ (13)

where *d*_*RV*_% is the percent RV differences, *RV*_*TPS*_ and *RV*_*m*_ represent the relative volumes calculated from the dose volumes of the TPS and the measurements, *D*_*s*_ is the starting dose of the dose range, *D*_*p*_ is the prescribed dose, and *t* denotes the measurement time.

## Results and Discussion

### Characteristics of gel dose response

**Fig 2:**
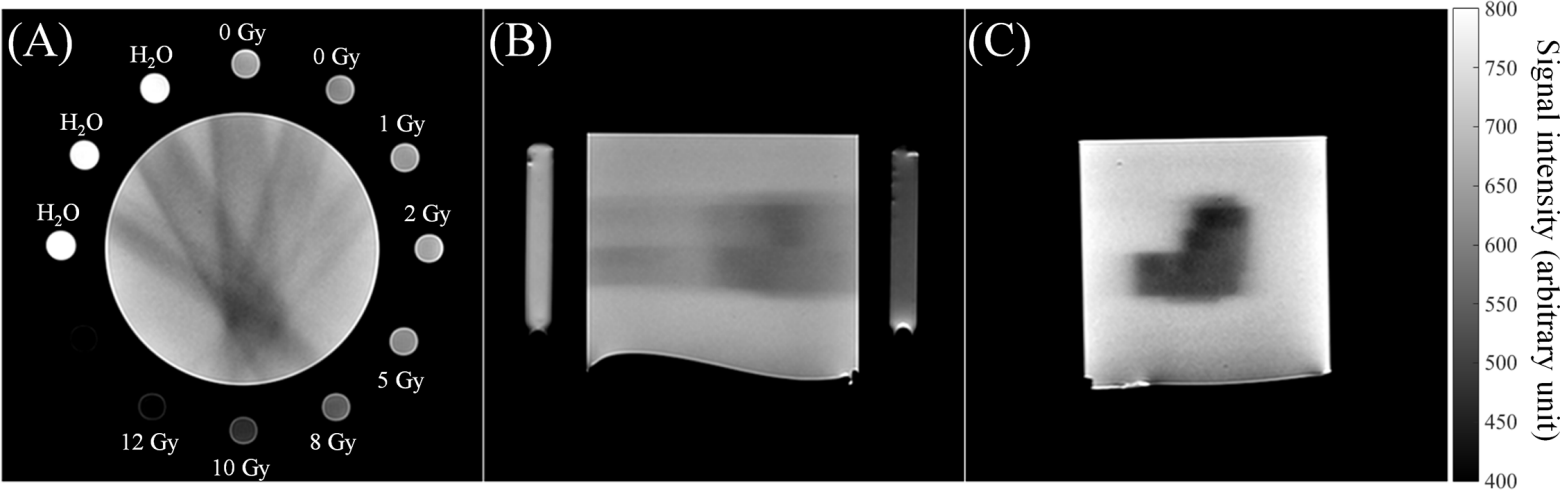
**(A) Axial, (B) sagittal, and (C) coronal T2-weighted images of the irradiated NIPAM gel phantom.** The poured materials and absorbed doses of the calibration tubes are labeled in (A).

**Fig 3:**
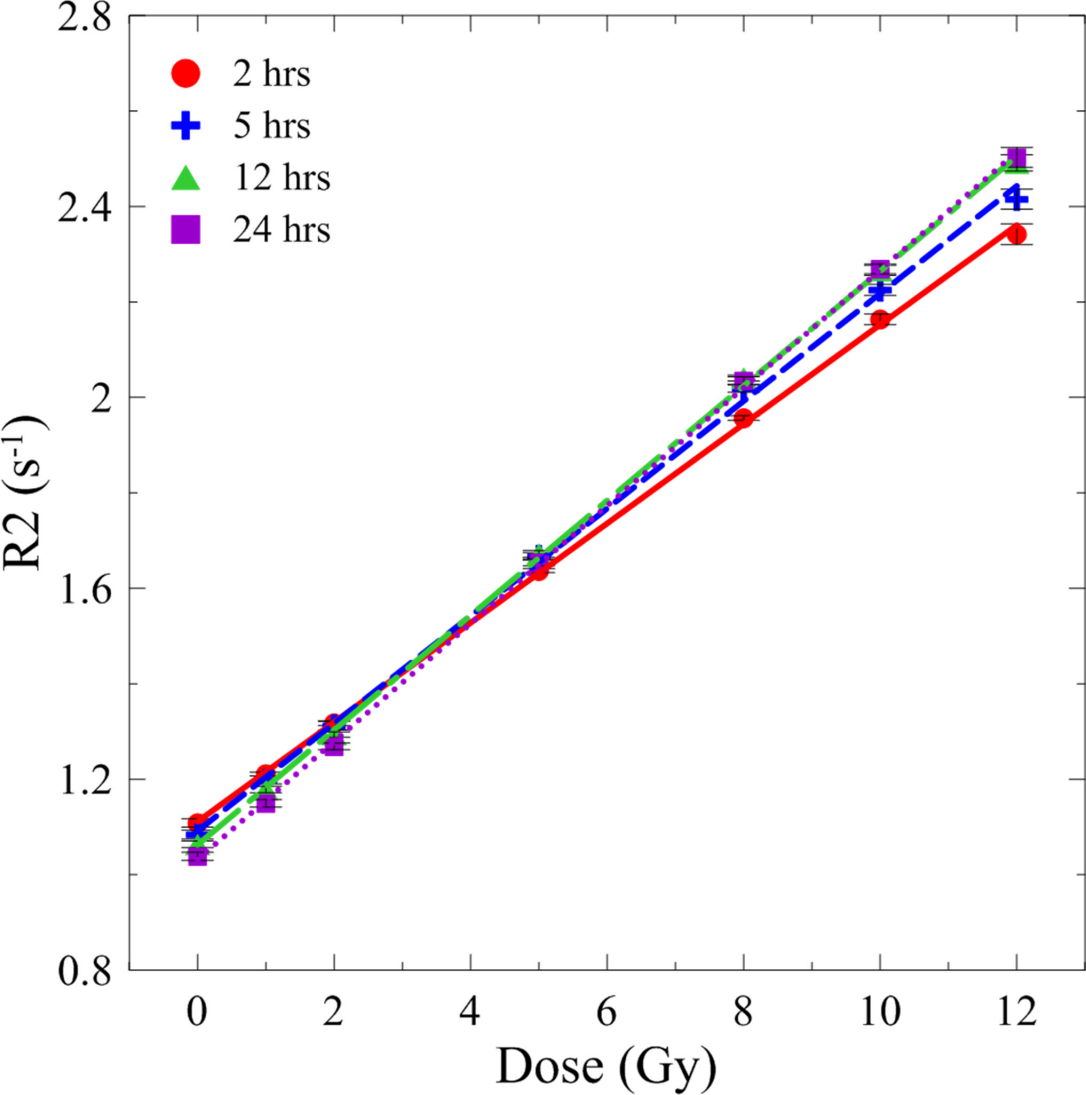
DRCs determined from 2, 5, 12, and 24 h T2-weighted images.

Fig 2 shows T2-weighted images of the irradiated NIPAM gel phantom from axial, coronal, and sagittal perspectives. The outer lanes in the axial images are 11 inserted tubes, three of which were filled with pure water and eight were filled with NIPAM gels for dose calibration. Two calibration tubes were unirradiated reference tubes (0 Gy) and six were irradiated tubes with absorbed doses of 1, 2, 5, 8, 10, and 12 Gy, separately. The contrast in the T2-weighted images can be interpreted as a dark image resulting from the dose absorbed by the NIPAM gel. The values corresponding to short T2 represent a high absorbed dose. The DRCs of the NIPAM gel dosimeters from 2, 5, 12, and 24 h measurements are shown in Fig 3. The fitting parameters and linearity of the DRC fits are listed in Table 1. The linearity of four DRCs was higher than 0.99. The coefficient of variance (CoV) of the slope among four DRCs was lower than 8%, indicated no significant sensitivity difference among the four readout time points. The temporal instability is mainly caused by continuous slight polymerization in normoxic polymer dosimeters after irradiation. The 12 and 24 h DRCs were highly matched for absorbed doses higher than 2 Gy.

**Table 1:** Standard errors of the parameters *a* and *b* (Δ*a* and Δ*b*), linearity of the DRC (*r*^2^), and the mean standard errors of the dose conversion from using the DRCs (mean Δ*D*).

Time (h)	*a* (s^−1^ Gy^−1^)	Δ*a* (s^−1^ Gy^−1^)	*b* (s^−1^)	Δ*b* (s^−1^)	*r*^2^	Mean Δ*D* (Gy)
2	0.104	0.0011	1.11	0.008	0.99	0.97
5	0.113	0.0017	1.09	0.012	0.99	0.94
12	0.12	0.001	1.06	0.007	0.99	0.91
24	0.124	0.001	1.03	0.007	0.99	0.74

### Uncertainty of dose conversion from using dose response curves

Table 2 lists the values and standard errors (Δ*a* and Δ*b*) of the fitting parameters *a* and *b* as well as the linearity of DRC (*r*^2^). The mean standard errors of the dose conversion using the DRCs (mean Δ*D*) are also listed. For all the measured time points, Δ*a* and Δ*b* are lower than 0.002 s^−1^ Gy^−1^ and 0.015 s^−1^, respectively, which indicates that the DRCs fit well. In addition, the mean Δ*D* of all the measured time points is lower than 1 Gy, which demonstrates that the dose conversion that uses the DRCs is accurate and reliable for all the measured time points.

**Table 2:** Means and standard deviations of pass rates of axial, coronal, and sagittal dose maps acquired at 2, 5, 12 and 24 h after dose delivery.

Time (h)	Pass rate (%)		
	Axial	Coronal	Sagittal
2	83.5 ± 0.9	90.6 ± 1.4	88.9 ± 0.5
5	85.9 ± 0.6	94.5 ± 0.7	93.4 ± 0.5
12	98.7 ± 0.3	97.6 ± 0.4	98.6 ± 0.9
24	98.5 ± 0.9	97.6 ± 0.6	98.9 ± 0.8

### Temporal stability of dose distributions in the cylindrical gel phantom

**Fig 4:**
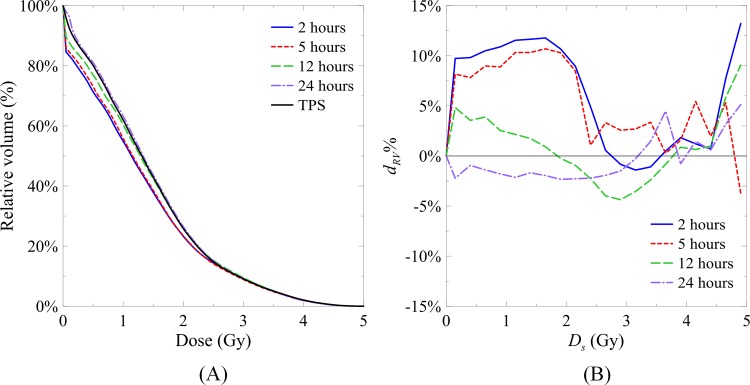
**(A) DVH calculated from TPS and 2, 5, 12, and 24 h dose volumes.** (B) Percent RV differences (*d*_*RV*_%) between the dose volumes from TPS and those from 2, 5, 12, and 24 h dose volumes.

Fig 4 illustrates the DVH and the percent RV differences between the dose volumes from the TPS and the measurements. When the starting dose was below 2.1 Gy, the differences between the 2 h and 5 h dose volumes were higher than 8%. When the initial dose exceeded 2.6 Gy, the differences decreased and were lower than 5%. By contrast, the differences between the 12 h and 24 h dose volumes were lower than 5% for volumes with an absorbed dose lower than 4 Gy. When the starting dose exceeded 4 Gy, the RV was lower than 2.1%, and the percent RV differences increased to over 5% for all the measured time points. The absolute mean percent RV differences between the 2, 5, 12, and 24 h dose volumes and that of the TPS were 6.48%, 5.77%, 2.78%, and 1.98%, respectively.

**Fig 5:**
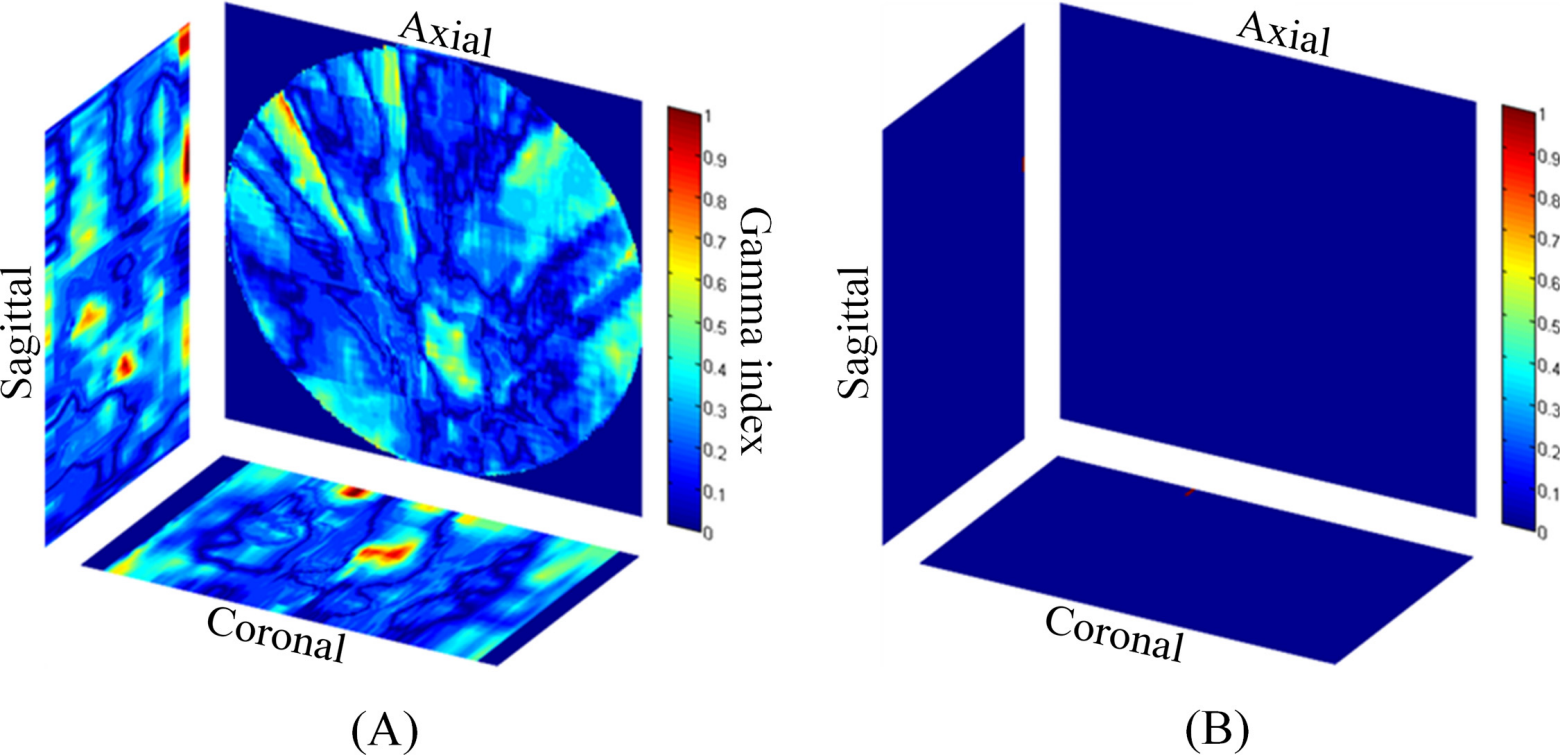
**(A) Axial, coronal, and sagittal gamma maps calculated from 24 h dose maps.** (B) Failed point maps, denoted in red.

Table 2 lists the mean pass rates and standard deviations of the three orthogonal dose maps calculated from the three gel phantoms. In the 2 h and 5 h dose maps, the pass rates were lower than 95%. The poor performance of the 2 h and 5 h measurements was mainly attributed to a continuous polymerization reaction surrounding the irradiated region where the absorbed dose was lower than 50% of the prescribed dose, as observed in the DVHs of the measured dose volumes and TPS (Fig 4). The pass rates of the dose maps increased as time elapsed and became stable after 12 h. The pass rates of 12 and 24 h dose maps were higher than 0.97, which is a common acceptable criterion for dose delivery in clinical practice. The gamma maps of the 24 h dose maps with 3%/3 mm criteria are shown in Fig 5. The gamma values of most of the dose points were lower than 1, thus passing the evaluation. A few failed points appeared at the edges of the sagittal and coronal dose maps. These failed points may be attributable to the image noise.

In the gamma evaluation, the mismatch between the dose maps from the NIPAM gel and TPS could also reduce the pass rates. The pass rates of a measured dose map converted from the R2 maps shown in Fig 2(A) were calculated using the original simulated map and the maps before and after the original map. On the basis of the 3%/3 mm criterion, the pass rates of the measured dose map to the previous, original, and subsequent simulated maps were 79.14%, 83.13%, and 81.47%, respectively. The results indicate that a poor mismatch among the dose maps strongly influences and degrades the pass rate of the dose maps. To ensure that the spatial location of the measured and simulated maps was as close as possible, the slice location and thickness of the MR scans were set to match the values used in the CT scans. On the basis of the MR-acquired dose map, the closest simulated dose map was used for gamma evaluation. The differences between the spatial location of the measured and simulated maps should be minimal.

De Deene et al. showed that most of the polymerization reactions occurred within the first 24 h after irradiation, with some lasting up to 30 days [20]. The stabilization of polymerization could be observed in the slope difference per hour of the DRC with time. The slope differences per hour between the DRCs of 2 h to 5 h, 5 h to 12 h, and 12 h to 24 h were 3×10^−3^, 1×10^−3^, and 3.3×10^−4^ s^−1^ Gy^−1^ h^−1^, respectively. After 22 h, the slope difference was reduced tenfold, which indicated that the polymerization had stabilized. However, the results of the DVHs showed that the NIPAM gel dosimeters with an absorbed dose higher than 2.8 Gy could be stabilized after 2 h post-irradiation. This finding confirmed that the polymerization reaction of the NIPAM gel dosimeters could be completed within a short post-irradiation period, as previously reported in [21]. This result was probably attributed to the intensive radicals produced at this dose level, which rapidly depleted most of the monomers in the NIPAM gels to form stable polymer structures for accurate dose readouts. By contrast, the NIPAM gels with an absorbed dose lower than 2.8 Gy retained a sufficient number of monomers to form relatively larger polymer structures than the initial ones. In addition, the production rate of radicals under such dose level was low. Consequently, stable polymer structures could not be formed instantly, which caused the errors in the 2 h DVH and the failure in the low-dose region during the gamma evaluation. In this case, the continuous reactions that occur in the NIPAM gels until 12 h post-irradiation are crucial to determine the low-dose region accurately. In this case, continuous reactions occurred in the NIPAM gels until 12 h postirradiation are crucial for accurately determining the low-dose region. In addition, the relationship between stabilization time and absorbed dose may be changed with different gel formulas, thus indicating the need for further study.

## Conclusion

In this study, the short-term stability of the NIPAM gels with MR readouts was evaluated using a clinical IMRT case. The results demonstrate that the NIPAM gel dosimeter with MR readouts could accurately provide the entire dose volume after a 12 h reaction time. In addition, volumes with an absorbed dose higher than 2.8 Gy can be rapidly obtained after a 2 h reaction time. It is therefore concluded that NIPAM gel dosimeters with MR readouts could be useful and convenient in dose verification of clinical IMRT.
